# Measurement of molecular biomarkers that predict the tumor response in estrogen receptor-positive breast cancers after dose-dense (biweekly) paclitaxel/carboplatin neoadjuvant chemotherapy

**DOI:** 10.18632/oncotarget.19686

**Published:** 2017-07-28

**Authors:** Teng Zhu, Fangping Xu, Liulu Zhang, Yifang Zhang, Ciqiu Yang, Minyi Cheng, Fulong Chen, Kun Wang

**Affiliations:** ^1^ Department of Breast Cancer, Cancer Center, Guangdong General Hospital & Guangdong Academy of Medical Sciences, Guangzhou 510080, China; ^2^ Department of Pathology, Cancer Center, Guangdong General Hospital & Guangdong Academy of Medical Sciences, Guangzhou 510080, China

**Keywords:** dose-dense, paclitaxel, carboplatin, ER-positive, biomarkers

## Abstract

The aim of this study was to determine the predictive value of the clinical and histopathological characteristics of estrogen receptor (ER)-positive patients treated with dose-dense paclitaxel/carboplatin neoadjuvant chemotherapy (NCT). Pathological complete response (pCR) and the change in tumor size between pre- and post-NCT were used to evaluate the tumor response.85 ER-positive breast cancer patients who were treated with dose-dense (biweekly) paclitaxel/carboplatin NCT were analyzed with respect to the expression of progesterone receptor (PgR), Tau, Ki67, human epidermal growth factor receptor 2 (HER2), and Bcl-2 by immunohistochemistry (IHC). These data were used to determine whether these biomarkers could predict the tumor response. A univariate analysis showed that the patients who tested positive for HER2 expression (56.00% vs 11.67%, p<0.01), negative for Tau expression (41.94% vs 14.81%, p=0.005), negative for Bcl-2 expression (46.43% vs 14.04%, p<0.01) and had smaller (≤2 cm) tumors (45.00% vs 18.46%, p=0.02) were associated with higher pCR rates. A multivariate analysis showed that a HER2-positive status (OR: 6.244; 95%CI: 1.734-22.487; p=0.005), Bcl-2-negative status (OR: 0.236; 95%CI: 0.064-0.869; p=0.030) and smaller (≤2 cm) tumor sizes (OR: 0.188; 95%CI: 0.046-0.767; p=0.020) are independent predictors of pCRs. The tumor sizes were significantly reduced in patients with HER2-positive, Tau-negative, Bcl-2-negative and high Ki67 index breast cancer. In conclusion, Bcl-2 negative, HER2-positive and smaller (≤2 cm) tumor sizes are independent predictors of pCR in ER-positive patients treated with dose-dense (biweekly) paclitaxel/carboplatin NCT. This study is registered with ClinicalTrials.gov (NCT0205986).

## INTRODUCTION

Chemotherapy is widely administered preoperatively for operable breast cancer [1]. Neoadjuvant chemotherapy is used to downstage cancers, increase the success rates of breast conservation therapy (BCT) and determine tumor chemosensitivity. The paclitaxel/carboplatin (PC) combination is an effective therapeutic option similar to paclitaxel/epirubicin therapy [2]. The PREPARE trial demonstrated that dose-dense neoadjuvant chemotherapy might result in higher pathological complete response (pCR) rates [3]. Our previous study showed that dose-dense PC neoadjuvant chemotherapy achieves high pCR rates in patients with HER2-positive, triple-negative, luminal cancer who previously showed low pCR rates (25.6%) [4].

Breast cancer is a heterogeneous disease that requires individualized treatment [5]. Overall, 75.4% of all cancers show estrogen receptor (ER) expression [6]. A study by European researchers showed that the pCR rate of patients with ER-positive tumors was markedly lower than that of patients with ER-negative tumors (12% vs 42%, respectively) [7], which implies that many of the patients who receive chemotherapy are not sensitive to the treatment. Thus, the identification of multiple biomarkers that can predict whether these patients can benefit from certain chemotherapy regimens is needed [8], and the predictive values of Tau and Bcl-2 remain unclear.

Tau protein interacts with tubulin to stabilize microtubules [9] and binds to the inner and outer surfaces of microtubules. Taxanes also bind to the same locations, and thus, Tau can obstruct the functions of taxanes [10]. Our previous study showed that Tau protein expression is correlated with breast cancer sensitivity in patients receiving taxane-based neoadjuvant chemotherapy [11]. The National Surgical Breast and Bowel Project (NSABP)-B 28 clinical trial demonstrated that 57% of ER-positive tumors show positive Tau expression compared with 15% of ER-negative tumors [12]. Substantial proportions of ER-positive/Tau-negative patients may benefit from taxane-based therapies. Previous studies have focused on the correlation between Tau expression and the efficiency of neoadjuvant chemotherapy (NCT) treatments that included taxanes [11].

Bcl-2 is localized to the mitochondrial outer membrane, and approximately 25%-50% of all primary breast cancers express high levels of Bcl-2 [13]. Bcl-2 is considered a favorable prognostic marker in multiple tumor subtypes, particularly ER-positive breast cancer [14]. However, the relationship between Bcl-2 expression and patient outcomes remains unclear. Chen et al. showed that patients with Bcl-2-negative tumors have higher pCR rates than patients with Bcl-2-positive diseases [15]. Fernández-Sánchez et al. did not observe a significant correlation between Bcl-2 expression and pCR rates [16].

Based on these data, the present study aimed to determine the predictive values of molecular biomarkers in ER-positive patients treated with dose-dense paclitaxel/carboplatin NCT.

## RESULTS

The clinical and pathological data of the patients are listed in Table 1. Of the 85 patients with ER-positive tumors, 81 were found to have stage II-III tumors, as determined by imaging studies. Stage I disease (T1cN0M0) was noted in four patients. 25 (29.41%) patients had HER2-positive tumors, and 60(70.59%) had HER2-negative tumors. 54 (63.53%) patients had Tau-positive tumors, and 31(36.47%) had Tau-negative tumors. In addition, 58 (68.24%) patients had Bcl-2-positive tumors, and 27 (31.76%) had Bcl-2-negative tumors.

**Table 1:** Clinical and pathological characteristics of 85 ER-positive breast cancer patients

	Characteristic	Number (%)
Age	≦35	7(8.24)
	35-55	64(75.29)
	≧55	14(16.47)
Menstrual status	Premenopause	56(65.88)
	Postmenopause	29(34.12)
Tumor size (cm)	≦2	20(23.53)
	>2, ≦5	59(69.41)
	>5	6(7.06)
Lymph node status	Positive	52(61.18)
	Negative	33(38.82)
Stage	I	4(4.71)
	II	70(82.35)
	III	11(12.94)
PgR status	Negative	4(4.71)
	Positive	81(95.29)
HER2 status	Negative	60(70.59)
	Positive	25(29.41)
Ki67 status	Negative	21(24.71)
	Positive	64(75.29)
Tau status	Negative	31(36.47)
	Positive	54(63.53)
Bcl-2 status	Negative	27(31.76)
	Positive	58(68.24)

ER= estrogen receptor, PgR= progesterone receptor, HER2= human epidermal growth factor receptor 2.

**Figure 1:**
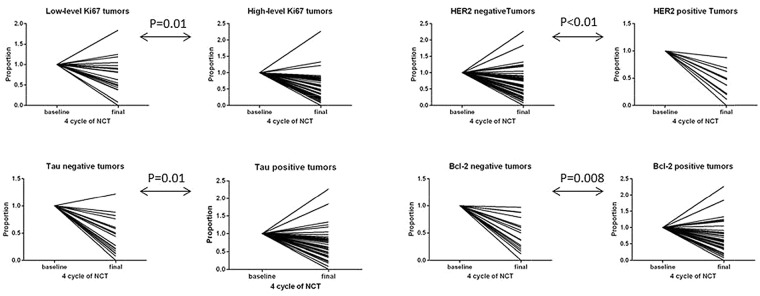
Radiologic size changes according to the status of molecular biomarkers

Of the 85 patients with ER-positive tumors, 21 achieved a pCR (24.71%). Table 2 shows that HER2-positive patients were more likely to achieve a pCR than HER2-negative patients (hazard ratio=9.636, 95%CI 3.158-29.408, p<0.01). Additionally, Tau-negative patients were more likely to achieve a pCR (hazard ratio=4.153, 95%CI 1.474-11.698, p=0.004) than Tau-positive patients, and patients with Bcl-2-negative tumors were more likely to achieve a pCR than patients with Bcl-2-positive tumors (hazard ratio=5.804, 95%CI 2.008-16.777, p=0.001). After four cycles of NCT, Tumor progression was noted in six patients, all of whom had HER2-negative/Tau-positive/Bcl-2-positive tumors. A significant reduction in tumor size was noted in patients with HER2-positive, Tau-negative, Bcl-2-negative and high Ki67 index (p<0.01, p=0.01, p=0.008, and p=0.01, respectively; Figure 1).

**Table 2:** Prediction of pCR by univariate analysis

Numbers of patients		pCR(%)	Odds ratio(95%CI)	P value
Menstrual status			0.794(0.285-2.209)	0.658
Premenopause	56	13(23.21)		
Postmenopause	29	8(27.59)		
Tumor size (cm)			3.614(1.226-10.652)	0.02
≦2	20	9(45.00)		
>2	65	12(18.46)		
Lymph node status			1.621(0.598-4.392)	0.341
Negative	33	10(30.30)		
Positive	52	11(21.15)		
PgR Status			0.984(0.097-9.997)	0.989
Negative	4	1(25.00)		
Positive	81	20(24.69)		
HER2 Status			9.636(3.158-29.408)	<0.01
Negative	60	7(11.67)		
Positive	25	14 (56.00)		
Ki67 Status			2.348(0.616-8.950)	0.202
Negative	21	3 (14.29)		
Positive	64	18 (28.13)		
Tau status			4.153(1.474-11.698)	0.005
Negative	31	13 (41.94)		
Positive	54	8 (14.81)		
Bcl-2 status			5.804(2.008-16.777)	0.001
Negative	28	13 (46.43)		
Positive	57	8 (14.04)		

A multiple logistic regression analysis showed that HER2 is the most significant predictor of a pCR. Patients with HER2-positive tumors were found to be 6.2-fold more likely to achieve a pCR than HER2-negative patients. Additionally, Bcl-2-negative tumors and negative axillary lymph nodes were identified as significant predictors of a pCR (Table 3).

**Table 3:** Multivariate logistic regression analysis of pathological complete response independent predictive factors

	Odds ratio	95%CI	P value
Tumor size (cm)			0.020
≤2	1	0.046-0.767	
>2	0.188		
HER2 Status			0.005
Positive	6.244	1.734-22.487	
Negative	1		
Tau status			0.109
Positive	0.364	0.106-1.254	
Negative	1		
Bcl-2 status			0.030
Positive	0.236	0.064-0.869	
Negative	1		

Twenty-five (29.41%) patients with HER2-positive tumors received trastuzumab concomitant with chemotherapy. We analyzed the association between molecular biomarkers and a pathologically complete response in 60 (70.59%) ER-positive/HER2-negative patients. The strength of this association was weaker in ER-positive/HER2-negative patients, and Tau expression was found to be significantly associated with a pCR (p=0.07). The strongest association between molecular biomarkers and a pathological complete response was observed in ER-positive/HER2-positive patients.

## DISCUSSION

Unlike disease-free survival (DFS) and overall survival (OS), the primary endpoint of a pCR can be rapidly reported as an approach for evaluating the efficacy of neoadjuvant chemotherapy [17]. The US Food and Drug Administration established Collaborative Trials in Neoadjuvant Breast Cancer (CTNeoBC), which revealed that the prognostic value of pCR rates is lower in hormone receptor (HR)-positive/HER2-negative tumors than in aggressive breast cancer subtypes (triple-negative and HER2-positive) [18]. However, these results should not be interpreted as implying that pCR is not a prognostic factor in luminal tumors. Symmans et al. [19] showed that the long-term prognosis for each breast cancer subtype is related to the residual cancer burden (RCB) after neoadjuvant chemotherapy. The data reported by these researchers confirmed that patients with pCR had a better prognosis than with those with other RCB classes, which were defined as follows using the RDB index: pCR (RCB-0), RCB-I, RCB-II and RCB-III. Thus, a pCR was considered the primary endpoint in this study. A correlation between tumor response and survival has been observed [20, 21]. However, quantifying the tumor response is required to establish a system for evaluating the prognosis after NCT. The change in tumor size between pre- and post-NCT might be a potential indicator of NCT efficacy in breast cancer.

Tau is an ER-induced gene, and 64% of ER-positive tumors were found to be positive for Tau expression in this study, which means that approximately 40% of ER-positive tumors do not show Tau expression. However, patients with ER-positive tumors are less sensitive to chemotherapy [22]. The identification of ER-positive patients who would benefit from chemotherapy is important. Paclitaxel is one of several cytoskeletal drugs that target microtubules and thereby block the progression of mitosis [23]. Tau protects microtubules from paclitaxel, which means that low Tau expression is associated with high paclitaxel sensitivity [11]. However, not all studies have noted a similar relationship between Tau expression and paclitaxel sensitivity [12, 24, 25]. This difference might be attributed to the anti-tumor activities of anthracycline-based drugs, which might interfere with that of paclitaxel. Therefore, we studied patients who used a non-anthracycline-based combination and found that low Tau expression is significantly associated with both high pCR rates and tumor size reductions (p=0.01 and p=0.004, respectively).

Bcl-2 has emerged as an important prognostic factor for the risk of breast cancer recurrence [26]. The precise predictive value of Bcl-2 is unclear. Most studies have shown that Bcl-2 overexpression is associated with a favorable prognosis [27, 28], although some trials have yielded different conclusions [29]. Our study showed that Bcl-2-negative patients can benefit from the neoadjuvant regimen discussed herein and that Bcl-2 is an independent predictor of a pCR. One reason for this inconsistency might be that Bcl-2 is also an ER-induced gene [30]. Fifty-seven (67.47%) of the patients included in this study had Bcl-2-positive tumors. In addition, patients with ER-positive tumors were found to be more likely to have a favorable prognosis, and thus, Bcl-2 cannot be an independent predictor of prognosis. Vaillant et al. found higher Bcl-2 expression in the luminal A subtype than in the luminal B subtype [31], which indicates that the predictive effects of Bcl-2 might be concealed by the favorable outcomes associated with the luminal A subtype. In addition, several studies have shown that Bcl-2 is a weak predictive marker of a pCR [16, 32]. This difference can be explained by the enrollment of patients with relatively advanced disease because the dose-dense PC regimen has been shown to have high anti-tumor activity.

ER-positive tumors exhibited continued reductions in size during the course of chemotherapy, and reductions in tumor size were rapidly achieved in ER-negative tumors during the first four cycles [33]. Several studies have shown that extended neoadjuvant chemotherapy is significantly associated with high pCR rates [34, 35]. High Ki67 indices have been linked to tumor size reductions (p=0.01), and there is no significant association between Ki67 expression and pCRs (p=0.179). However, we hypothesize the existence of a significant association between Ki67 expression and pCRs after six to eight cycles of PC in ER-positive tumors, which warrants further exploration. In addition, further investigation is needed to collect survival data after a prolonged follow-up period, and additional biomarkers are needed to predict outcomes in patients with ER-positive tumors.

In summary, HER2-positive/Tau-negative/Bcl-2-negative tumors are associated with higher pCR rates. This study identified HER2 and Bcl-2 as independent predictors of a pCR through a logistic regression analysis, and significant reductions in tumor size were noted in HER2-positive/Tau-negative/Bcl-2-negative tumors with a high Ki67 index.

## MATERIALS AND METHODS

Between January 2012 and June 2014, 85 patients with operable ER-positive breast cancer were enrolled in this study. Pathological confirmation of each tumor was performed by core needle biopsy. The absence of metastatic disease was confirmed by computed tomography, whole-body bone scans or PET/CT. Ultimately, patients with primary operable tumors (cT1c-3N0-1M0) were enrolled in the study. Ultrasound screening was performed for staging evaluation prior to neoadjuvant chemotherapy administration and surgery. Patients aged 18 to 70 years with PS scores of 0–1, without cardiac disease, and with normal bone marrow (absolute neutrophil count≥1.5×10^9^/L, platelets≥100×10^9^/L, and hemoglobin≥100 g/L), liver (total bilirubin AST and ALT levels≤1.5-fold higher than the upper limit of normal), and renal function (creatinine≤175 μmol/L) were considered eligible for the study.

The cutoff values for estrogen receptor (ER), progesterone receptor (PgR), HER2 and Ki67 positive expression were set to those described in our previous study [4]. The cutoffs for estrogen receptor (ER) and progesterone receptor (PgR) positivity were both 1%, as determined by nuclear staining, and the cutoff value for the Ki67 proliferation index was 15%. With respect to HER2 testing, immunohistochemistry (IHC) 3+ results or fluorescence *in situ* hybridization (FISH)-amplified results were considered to indicate HER2 positivity, whereas IHC0 or 1+ results or ISH non-amplified results were considered to indicate HER2 negativity, and borderline IHC results were subjected to ISH testing by counting the additional cells, ISH retesting, or reflex testing using a validated IHC method. The cytoplasmic staining intensity of Tau protein in the normal mammary epithelium was used as a reference for scoring. Cancer cells without Tau staining were given a score of 0, cancer cells with less Tau staining than the normal mammary epithelium were given a score of 1, cancer cells with staining levels similar to those of the normal mammary epithelium were given a score of 2, and cancer cells with higher staining levels than the normal mammary epithelium were given a score of 3. Cells with scores of 0 and 1 were considered Tau-negative cells, and cells with scores of 2 and 3 were considered to have Tau positive expression [36]. The cytoplasmic immunoreactivity for Bcl-2 was determined based on the percentage of positively stained cancer cells, and the cutoff value was set to 30% [37].

### Treatment

The details regarding the specific treatment methods investigated in this study have been reported elsewhere [4]. All of the patients received four cycles of dose-dense (biweekly) PC at a dose of 175 mg/m^2^ paclitaxel and carboplatin (AUC=5) on day 1. Patients with HER2-positive disease simultaneously received trastuzumab treatments (initial dose of 6 mg/kg followed by biweekly doses of 4 mg/kg). All patients received granulocyte colony-stimulating factor (G-CSF) on days 2 and 3 at a dose of 300 μg per day. Ultrasound screening was performed for staging prior to treatment.

### Response assessment

The primary endpoint was a pathologically complete response (pCR), and the secondary endpoint was the change in tumor size pre- and post-NCT. A pCR (ypT0N0) was defined as the absence of both the primary tumor and axillary lymph node metastasis.

### Statistics

The X^2^ test was used to correlate the various clinicopathological factors with pCR. Predictors of the change in tumor size pre- and post-NCT were evaluated using Student's t test. A multivariable analysis based on logistic regression was performed to evaluate the correlations between potential predictive factors and pCR. A previous study revealed that the pCR rate can range from 6% to 38% based on the subtype [38]. We assumed that the patients would achieve pCR rates between 10% and 40% and that we needed to enroll 79 patients. All p values were two-tailed, and p<0.05 was considered to indicate statistical significance. The SPSS Software package (version 23.0; SPSS Company, Chicago, IL, USA) was used for these analyses.
